# Theoretical study of the H/D isotope effect of CH_4_/CD_4_ adsorption on a Rh(111) surface using a combined plane wave and localized basis sets method

**DOI:** 10.1039/d0ra10796d

**Published:** 2021-03-10

**Authors:** Hiroki Sakagami, Masanori Tachikawa, Takayoshi Ishimoto

**Affiliations:** Graduate School of Nanobioscience, Yokohama City University 22-2 Seto, Kanazawa-ku Yokohama 236-0027 Japan; Graduate School of Data Science, Yokohama City University 22-2 Seto, Kanazawa-ku Yokohama 236-0027 Japan; Department of Applied Chemistry, Graduate School of Advanced Science and Engineering, Hiroshima University 1-3-2 Kagamiyama Higashihiroshima 739-8511 Japan tishimo@hiroshima-u.ac.jp; Division of Materials Model-Based Research, Digital Monozukuri (Manufacturing) Education and Research Center, Hiroshima University Higashi-Hiroshima 739-0046 Japan

## Abstract

We analysed the H/D isotope effect of CH_4_/CD_4_ adsorption on a Rh(111) surface using our combined plane wave and localized basis sets method, that we proposed for the consideration of delocalized electrons on a surface and the quantum effect of protons (deuterons) in metal–molecule interactions. We observed that the adsorption distance and energy of CD_4_ were larger and lower than those of CH_4_, respectively. This is in reasonable agreement with the corresponding experimental results of cyclohexane adsorption. We clearly found that the trend of the H/D isotope effect in the geometrical and energetic difference was similar to that of the hydrogen-bonded systems.

## Abstract 2

Using our CPLB method, we elucidate that the adsorption distance and adsorption energy of CH_4_ on the Rh(111) surface are shorter and larger than those of CD_4_, which is in reasonable agreement with the corresponding H/D isotope trend in experiments.

## Graphical abstract

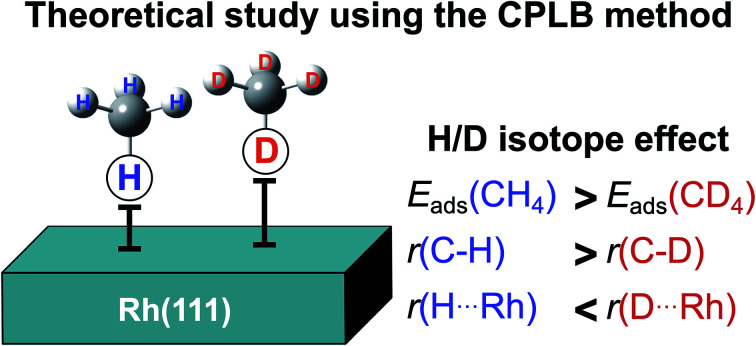

## Introduction

The behaviour of atoms and molecules on various surfaces has attracted attention in various fields, including materials science, material physics, and catalytic chemistry. In particular, the interaction between hydrogen (H) and transition metals is a crucial factor in chemical and physical processes. The H in the molecules that are adsorbed on metal surfaces determines important phenomena for not only fundamental physics but also for catalytic activity.^1,2^ It has been reported that the differences in adsorption energy, diffusion coefficient, and catalytic activity depend on various metals and surface orientations.^3–5^ Despite these studies, the interaction mechanism between H and metal surfaces is still not clearly understood due to the difficulty in the direct analysis of H on the metal surface from only the experimental side.

The interaction between H and metal surfaces is often analysed by deuterium (D) substitution for H. This helps in understanding the details of molecular adsorption on metal surfaces from the viewpoint of H/D isotope effects. Thus, understanding the H/D isotope effect is one of the key factors for surface design. For example, using a high-vacuum microbalance, Jin *et al.* observed that the adsorption energy of D_2_ (0.302 eV) is larger than that of H_2_ on the surface of a Pd–Pt alloy.^6^ Zheng *et al.* observed that the diffusion coefficient of H (0.027 cm^2^ s^−1^) is larger than that of D on Pd(111) surfaces.^4^ These experimental results clearly indicate that the adsorption of D on metal surfaces is stronger than that of H. Moreover, H/D isotope effects in the chemical reactions of the adsorbed molecules on metal surfaces, such as oxygen reduction and hydrogen exchange reactions, were reported.^7–9^ However, it is difficult to directly analyse the H/D isotope effects in detail in the structural parameters of the adsorbed molecules on the metal surfaces.

Recently, Koitaya *et al.* observed the H/D isotope effects on the adsorption energy and adsorption distance of the molecules of cyclohexane (C_6_H_12_) and its deuterium substituent (C_6_D_12_) on Rh(111) surface.^10^ To the best of our knowledge, this is the first report discussing the H/D isotope effects on the geometrical differences of molecules adsorbed on a metal surface. Interestingly, they clearly elucidated that the adsorption energy of C_6_H_12_ was 0.084 eV higher than that of C_6_D_12_. This result shows an opposite trend of the H/D isotope effects on H/D atomic adsorption on the metal surfaces. However, the mechanism of this inverse isotope effect is still unclear. Therefore, it is necessary to understand the mechanism of the geometrical H/D isotope effects of adsorbed molecules on metal surfaces.

It is well known that the electronic structure calculation is one of the most powerful approaches for elucidating the detailed mechanism of adsorbed molecules on the metal surfaces. The plane wave (PW) approach using periodic boundary conditions (PBCs) is an effective tool for understanding physical and chemical phenomena on the metal surfaces. There are also many studies on H adsorption on the metal surfaces.^11–14^ Since the nuclear quantum effects (NQEs) of protons and deuterons are treated as a difference of nuclear mass in the framework of the Born–Oppenheimer approximation in the conventional electronic structure calculation, the H/D isotope effects are discussed using the vibrational zero-point energy under harmonic oscillator approximation.^15^ However, reproducing the geometrical differences under the above treatment of protons and deuterons is difficult. Thus, it is essential to analyse the geometrical differences in the metal–hydrogen interaction that are induced by the difference in the NQEs of protons and deuterons. Although the direct treatment of the NQEs of protons and deuterons is important in electronic structure calculations for discussing the H/D isotope effects, PW embedded NQEs have not yet been developed. It is also difficult to describe small geometrical and electronic structure differences in H and D by the PW approach.

For the direct treatment of the difference of the NQEs of protons and deuterons, we developed multicomponent molecular orbital and density functional theory (MC_MO and MC_DFT) approaches, which are based on localized orbitals (LOs).^16–18^ Using these approaches, it is possible to describe the geometrical and electronic structure changes that are induced by H and D.^17,19^ Although these approaches are useful in investigating the H/D isotope effects for adsorbed molecules on metal surfaces, the calculation of large systems, such as those defined by PBCs, is not realistic due to high computational cost. Moreover, the addition of LO-based MC_MO and MC_DFT to the PW approach is challenging due to the high cost of program modification and computational accuracy. Thus, to achieve sufficient accuracy for analysing the H/D isotope effects for adsorbed molecules on metal surfaces, we proposed the combined plane wave and localized basis sets (CPLB) method.^20^ In the CPLB method, the electronic structure of the surface system is calculated using the PW approach. Conversely, a highly accurate LO calculation that is based on the MC_MO or MC_DFT approach is used for cluster model to describe the NQEs of protons and deuterons directly. The combination of both the PW- and LO-based approaches facilitates a detailed analysis of the H/D isotope effects for molecular adsorption on metal surfaces. We have already reported the efficiency of the CPLB method in analysing the H/D isotope effects of H/D atomic adsorption on the Pd(111) surface.^21^

In this study, we calculated the CH_4_/CD_4_ adsorption on the Rh(111) surface, which is a model of C_6_H_12_/C_6_H_12_ adsorption on Rh(111) surface experimentally,^10^ using the CPLB method to analyse and understand the geometrical and energetic changes induced by the H/D isotope effect.

## CPLB method

**Fig. 1:**
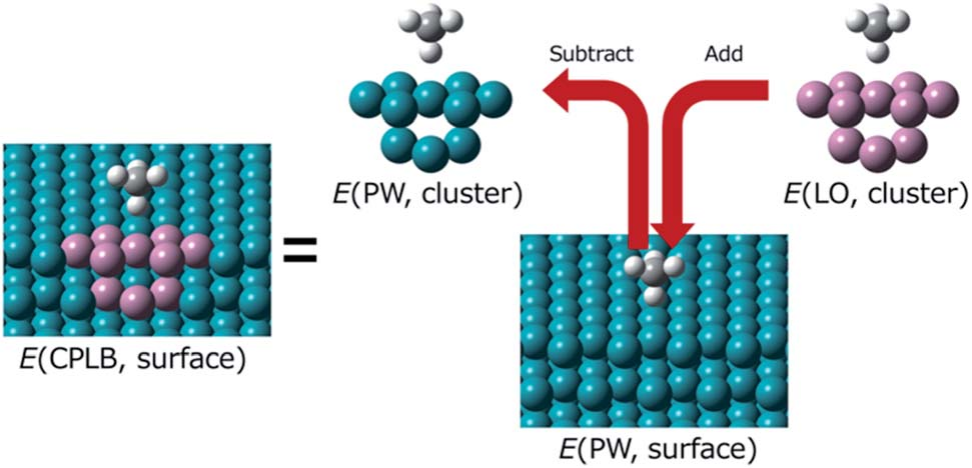
Schematic illustration of the CPLB approach for CH_4_ adsorption on a Rh(111) surface. The green and pink balls are Rh atom. These were treated using the PW and LO methods, respectively.

Herein, we explain the framework of our proposed CPLB method. The CPLB method is developed by extending it to a periodic system that is based on the ONIOM-like scheme.^22^ The procedure for the calculation of adsorption energy for the CH_4_ adsorption on the Rh(111) surface is illustrated in Fig. 1. The total energy of the surface system (*E*(PW, surface)) consisting of the Rh(111) surface with adsorbed CH_4_ was calculated using the PW-based electronic structure calculations. The total energy of adsorbed CH_4_ on the Rh cluster model, *i.e.*, *E*(PW, cluster) and *E*(LO, cluster), were calculated using both the PW- and LO-based electronic structure calculations, respectively. Based on these values, the total energy of the surface (*E*(CPLB, surface)) is calculated by the CPLB method using eqn (1), which provides sufficient accuracy by including both delocalized electronic structure in PW and accurate metal–hydrogen interaction in the LO. In addition, by selecting the appropriate calculation method as PW or LO calculation, it is also possible to further analyse. In this study, we calculated these systems including the NQEs of proton and deuteron by selecting the MC approach for the LO calculation.1
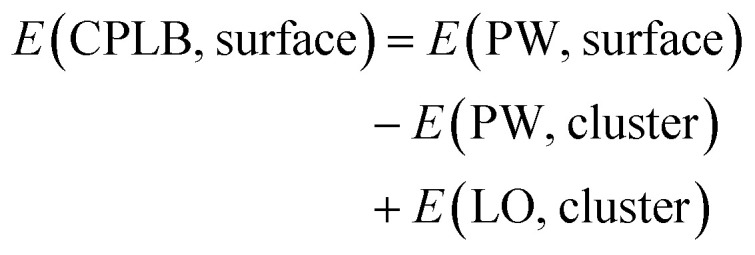
The grad *E*(CPLB, surface) can be obtained by using the gradient with respect to the nuclear coordinates for each energy, as follows:2
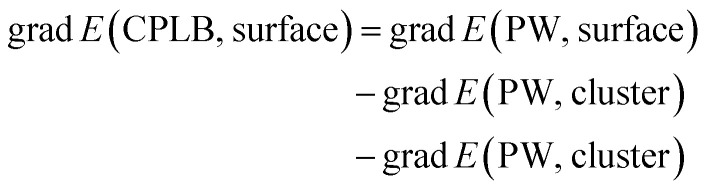


Using this scheme, we developed a geometry optimisation algorithm for the CPLB method. The adsorption energy and various other properties of CH_4_ were also calculated using the same procedure.

## Computational details

The computational details of each calculation are as follows: for the PW-based electronic structure calculations, we used the Vienna *ab initio* simulation package (VASP)^23,24^ with the projector-augmented wave method.^25,26^ The Perdew–Burke–Ernzerhof approximation with a generalized gradient approximation^27^ was used for the exchange and correlation functionals. The cut-off energy was set to 800 eV. The structure of the Rh(111) surface was modelled based on three atomic layers with 16 atoms in each layer since the energy difference between 3 and 4 or 5 atomic layers was less than 0.005 eV for CH_4_ adsorption. In the surface model calculation of the PBCs, 4 × 4 × 1 *k*-points were sampled using the Monkhorst–Pack grid method.^28^ For the LO-based electronic structure calculations, the modified Gaussian16 program package^29^ embedded with the MC_DFT method^18^ was used to take into account the NQEs of protons and deuterons. For electrons, the LanL2DZ basis set with an effective core potential^30–32^ was used for Rh atoms, and the 6-31G(d,p) basis set was used for other atoms in the APFD method.^33^ For the nucleus, the single s-type Gaussian-type function, exp{−*α*(*r* − *R*)^2^}, was used for each protonic and deuteronic basis function. The *α* values for protons and deuterons were set to the standard values of 24.1825 and 35.6214, respectively.^34^

The on-top site on the Rh(111) surface was found to be the most stable site for CH_4_ adsorption compared to face-centered cubic and hexagonal close-packed sites. Based on these results, a H/D atom in CH_4_/CD_4_ was placed at the on-top site on the Rh(111) surface as initial structure obtained by conventional PW approach. The geometry of this system was optimised as follows. First, the position of the surface atoms in the top (1st) layer was optimised using the PW approach. Then, CH_4_ was placed at the on-top site, and CH_4_ and Rh atoms in the top layer were optimised using the PW approach. Finally, the atomic coordinates of CH_4_/CD_4_ were optimised using the CPLB method including the MC_DFT approach as an LO part under the condition of fixed Rh atoms on the surface.

## Results and discussion

**Fig. 2:**
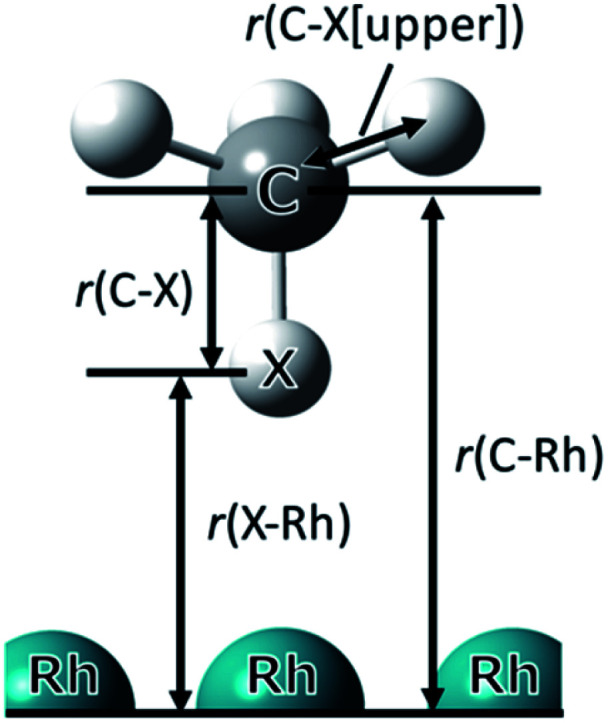
Optimised adsorption structure of CH_4_ on Rh(111) surface. The symbols used in Table 1 are also illustrated here. X indicates H or D in CH_4_/CD_4_.

In this study, we show and discuss the results of the optimised geometry parameters, adsorption energy, and charges for CH_4_/CD_4_ adsorption on Rh(111) surface. The optimised parameters for adsorbed CH_4_/CD_4_ are listed in Table 1. The symbols used in Table 1 are illustrated in Fig. 2.

**Table 1:** The optimised parameters (Å) for adsorbed CH_4_/CD_4_ at the on-top site on Rh(111) surface using the CPLB method. X indicates H or D in CH_4_/CD_4_. *r*(C–X [upper]) is the average value of three C–H/C–D distances pointing upward

	CH_4_	CD_4_	Δ(CD_4_−CH_4_)
*r*(C⋯Rh)	3.190	3.226	0.036
*r*(X⋯Rh)	2.071	2.111	0.040
*r*(C–X)	1.119	1.115	−0.004
*r*(C–X[upper])	1.112	1.105	−0.007

In Table 1, Δ(CD_4_−CH_4_) is the difference in the optimised parameters between CH_4_ and CD_4_. A positive value indicates that the bond distance of CD_4_ was longer than that of CH_4_. Since *r*(C⋯Rh) is the distance between C the nearest neighbour (just below) Rh atom, its value was considered as the adsorption distance of the CH_4_/CD_4_ molecule on the Rh(111) surface. The *r*(C⋯Rh) of CD_4_ was longer than that of CH_4_.

Looking at the geometrical parameters of *r*(X⋯Rh), the *r*(D⋯Rh) in the CD_4_ adsorption was longer than that in the CH_4_ adsorption as well as *r*(C⋯Rh). Furthermore, both *r*(X⋯Rh) and *r*(C⋯Rh) are shorter than the values using the conventional PW approach (2.322, 3.429 Å), because of the NQEs. These *r*(C⋯Rh) and *r*(X^⋯^Rh) geometrical H/D isotope effect for the CH_4_/CD_4_ adsorption showed the similar trend as the difference of adsorption distance of C_6_H_12_/C_6_D_12_ that is observed in the experiments by Koitaya *et al.*^10^ In contrast, the *r*(C–D) of CD_4_ was shorter than that of CH_4_. Compared to the C–X distance in isolated CH_4_/CD_4_ (1.113/1.106 Å), which is calculated by the MC_DFT method, we clearly observed the same trend of geometrical H/D isotope effect. The H/D isotope effect that the covalent bond distance of the C–D bond is shorter than that of the C–H bond was due to the anharmonicity of the potential. Furthermore, both *r*(C–D) and *r*(C–H) are longer than the C–H/C–D distance in isolated CH_4_/CD_4_ owing to the interaction between CH_4_/CD_4_ and the Rh(111) surface. These results indicate that the geometrical changes due to D substitution for H were opposite in the *r*(C⋯Rh) and covalent *r*(C–X) cases and H/D atomic adsorption on the Pd(111) surface.^21^ The difference in *r*(C⋯Rh) is similar to the Ubbelohde effect observed in typical hydrogen-bonded systems.^35,36^ Hence, the C⋯Rh interaction was considered to be different from H/D atomic adsorption and essentially the same as the typical hydrogen-bonded systems.^37^ In addition, this result indicates that the interaction between CD_4_ and Rh(111) was weaker than in CH_4_ case.

Further, we analysed the adsorption energy of CH_4_/CD_4_ on the Rh(111) surface, and the results are listed in Table 2. The adsorption energy of CH_4_ on the Rh(111) surface was defined as follows;

3 *E*_ads_ = *E*_tot_ − (*E*_surf_ + *E*_CH_4__)

where *E*_ads_ is the adsorption energy of CH_4_ on the Rh(111) surface, *E*_tot_ is the total energy of CH_4_ adsorbed on the Rh(111) surface, and *E*_surf_ and *E*_CH_4__ are the total energies of the Rh(111) surface model and CH_4_ molecule, respectively. A negative *E*_ads_ value indicates stable adsorption of CH_4_ on the Rh(111) surface. The *E*_ads_ of CD_4_ was lower than that of CH_4_. The *E*_ads_ difference of CH_4_/CD_4_ showed the same trend as C_6_H_12_/C_6_D_12_ adsorption, which was observed in the experiments performed by Koitaya *et al.*^10^ We also confirmed that CD_4_ has a weaker interaction with the Rh(111) surface than CH_4_.

**Table 2:** Adsorption energy (*E*_ads_) (eV) of CH_4_/CD_4_ on Rh(111) surface

	CH_4_	CD_4_	Δ(CD_4_−CH_4_)
*E*_ads_(CPLB, surface)	−0.402	−0.396	0.006

Finally, we performed a natural bond orbital (NBO) analysis^38^ during the LO calculation. The NBO charges of the interacting H/D atom with the Rh(111) surface and the total charges of CH_4_/CD_4_ are listed in Table 3. Different NBO charges were observed in the interacting H/D atom and the total charges of CH_4_/CD_4_. The charges around the H atom (0.249) were larger than those around the D atom (0.242). This observation suggests that the number of electrons around the proton is lower than that around the deuteron. In the CH_4_/CD_4_ molecule, the total charge around the CH_4_ molecule (0.053) was larger than around CD_4_ (0.043), which showed a similar trend of interacting H/D atoms. In fact, the magnitude of the dipole moment of CH_4_ (0.465 D) was slightly larger than that of CD_4_ (0.415 D) expectedly. These results suggest that the number of electrons transferred to the Rh(111) surface was larger in CH_4_ than that in CD_4_, and CH_4_ has a stronger interaction with the Rh(111) surface than CD_4_.

**Table 3:** The NBO charges of the interacting H/D atom in CH_4_/CD_4_ with Rh(111) and the total charges of CH_4_/CD_4_

	CH_4_	CD_4_	Δ(CD_4_−CH_4_)
Interacting H/D	0.249	0.242	−0.008
CH_4_/CD_4_	0.053	0.049	−0.004

## Conclusions

We studied CH_4_/CD_4_ adsorption on Rh(111) surface to analyse and understand the H/D isotope effect on the molecules that are adsorbed on metal surfaces. We developed the CPLB method for highly accurate calculation for analysis of molecular adsorption on metal surfaces, which is difficult to calculate using conventional methods. Consequently, we observed the H/D isotope effects in terms of adsorption distance, adsorption energy, and charge. Our results suggest that the mechanism of molecular adsorption on metal surfaces differs from that of atomic adsorption. Furthermore, the molecular adsorption shows the H/D isotope effects that are similar to those observed in conventional hydrogen-bonded systems. Based on these observations, we confirm that our developed CPLB approach is effective in reproducing the H/D isotope effects on CH_4_/CD_4_ adsorption on metal surfaces. The extension to other atomic or molecular adsorption on various types of surface would be possible in the framework of CPLB approach. Moreover, the CPLB approach is expected to be a powerful tool for analysing H/D isotope effects on various phenomena, such as adsorption, desorption, absorption, and catalysis, that involve atoms/molecules on heterogeneous systems such as surfaces and bulk systems.

## Conflicts of interest

There are no conflicts to declare.

## Supplementary Material
